# Lovastatin Reduces Stemness via Epigenetic Reprograming of *BMP2* and *GATA2* in Human Endometrium and Endometriosis 

**DOI:** 10.22074/cellj.2016.3894

**Published:** 2016-12-21

**Authors:** Mahdieh Taghizadeh, Mehrdad Noruzinia

**Affiliations:** Department of Medical Genetics, Faculty of Medical Sciences, Tarbiat Modares University, Tehran, Iran

**Keywords:** Endometriosis, Lovastatin, Epigenetics, Stemness

## Abstract

**Objective:**

The stem cell theory in the endometriosis provides an advanced avenue of targeting these cells as a novel therapy to eliminate endometriosis. In this regard, studies showed that lovastatin alters the cells from a stem-like state to more differentiated condition and reduces stemness. The aim of this study was to investigate whether lovastatin treatment could influence expression and methylation patterns of genes regulating differentiation of endometrial mesenchymal stem cells (eMSCs) such as *BMP2*, *GATA2* and *RUNX2* as well as eMSCs markers.

**Materials and Methods:**

In this experimental investigation, MSCs were isolated from endometrial and endometriotic tissues and treated with lovastatin and decitabin. To investigate the osteogenic and adipogenic differentiation of eMSCs treated with the different concentration of lovastatin and decitabin, *BMP2*, *RUNX2* and *GATA2* expressions were measured by real-time polymerase chain reaction (PCR). To determine involvement of DNA methylation in *BMP2* and *GATA2* gene regulations of eMSCs, we used quantitative Methylation Specific PCR (qMSP) for evaluation of the *BMP2* promoter status and differentially methylated region of *GATA2* exon 4.

**Results:**

In the present study, treatment with lovastatin increased expression of *BMP2* and *RUNX2* and induced *BMP2* promoter demethylation. We also demonstrated that lovastatin treatment down-regulated *GATA2* expression via inducing methylation. In addition, the results indicated that CD146 cell marker was decreased to 53% in response to lovastatin treatment compared to untreated group.

**Conclusion:**

These findings indicated that lovastatin treatment could increase the differentiation of eMSCs toward osteogenic and adiogenic lineages, while it decreased expression of eMSCs markers and subsequently reduced the stemness.

## Introduction

Endometriosis is a non-cancerous gynecological disorder characterized by the presence of endometrial glands and stromal cells outside the uterine cavity (1). It can be considered as the obvious cause of disability in the women with the reproductive ages affecting 6-12% of the asymptomatic women, approximately 71-78% of women suffering chronic pelvic pain and up to 5% of the infertile women (2,4). 

Pathogenesis of endometriosis includes metaplastic alteration of epithelial cells in the peritoneal surface (5), retrograde of endometrial cells, immunological insufficiency, genetics and epigenetics (6,7), as well as hormone disruption (8). Highly embraced hypothesis for the endometriosis development is retrograde menstruation (9). It was found that women with endometriosis have a considerable amount of basalis endometrium in their menstrual debris than those without endometriosis (10), more likely because of the excessive uterine peristaltic contractions in women suffering this disease (11). The importance of basalis layer of endometrium in the endometriotic lesion development can be related to the large numbers of stem cells in this area (12). In this respect, some studies also revealed that the stem cell theory has a significant role in the endometriosis pathogenesis (13, 14).

In addition, recent medications were found to have severe side-effects for treating endometriosis. Therefore, topnotch and effective treatments for endometriosis are required. The main mode of action for all of the present medications in treating pains, associated with endometriosis, is mostly caused by suppression of the implants proliferation (15, 16). The theory of stem cell in endometriosis began the last advanced avenue in the targeting these cells as cutting-edge therapy (17). 

In this respect, lovastatin shifts the cells from a stem-like state to more differentiated condition and reduces the stemness (18). Furthermore, lovastatin is effective in the suppression of cell proliferation and angiogenesis in an experimental model of endometriosis (19). In this line, lovastatin function via modulating DNA methyltransferase (DNMT) activity, altering methylation of gene promoters, and consequently regulating mRNA expression in the various malignancies (18, 20).

On the other hand, activity of DNMTs, the enzymes that catalyze addition of methyl groups to cytosine residues in DNA, is elevated in the ectopic endometrium compared to the normal control (21). DNMT inhibitors have profoundly been examined as the promising novel drugs for endometriosis treatment (22- 24). Recently, decitabine and 5-azacytidine have been introduced into the clinical trial experiment (25), but it was found that DNMT inhibitors cause considerable toxicity. In addition, they interfere with protein translation procedure through incorporating into RNA (26, 27). Because of this reason, drugs like statins, demonstrating DNMT inhibitory function with no toxic side-effect, would open up a new horizon regarding the novel advancement in the disease treatment.

Some investigations revealed that lovastatin treatment leads to demethylation of the *BMP2* promoter, up-regulation of the *BMP2* mRNA and activation of BMP signaling pathway. Consequently, these alterations induce colorectal cancer (CRC) cell differentiation and reduce proliferation of the respective cells (18, 28).

Moreover, BMP pathway, particularly *BMP2* plays a crucial role in the pathogenesis of endometriosis (29). *BMP2*, a tumor growth factor (TGF) superfamily member, acts down- stream of PGR and is essential for the stromal cell differentiation and decidualization in both mouse and human endometrium (30). Furthermore, Aghajanova et al. (31) found that BMP-2 can promote osteogenic differentiation of the human endometrial stem cells.

In this study, we initially set out to determine (1) whether lovastatin treatment influences methylation status of the *BMP2* promoter as well as mRNA expression of the respective gene and (2) whether lovastatin can also alter the expression level of other genes playing pivotal role in differentiation and proliferation potential of endometrial mesenchymal stem cells (eMSCs), such as *GATA2* and *RUNX2* (32, 33). Additionally, we then evaluated the effects of lovastatin on the endometrial stem cell markers derived from the patient and normal individuals.

## Materials and Methods

### Patients

This experimental investigation was approved by the Institutional Review Board of the Faculty of Medicine at Tarbiat Modares University in Iran. Endometrial and endometriotic tissues were obtained from six patients (endometrial tissues from three patients; endometriosis samples from three patients) at Obstetric Gynecology Department of Sarem Women Hospital (Tehran, Iran). The patients were undergone hysterectomy and laparoscopy for benign pathologies and written informed consent was also received from the participants. The surgery was performed irrespective of the day of patient’s menstrual cycle. The exclusion criteria were any endometrial abnormality (e.g. polyps, hyperplasia or cancer), administration of the hormonal treatment and gonadotropin- releasing hormone (GnRH) agonist therapy. Additionally, this study was performed according to the Helsinki declaration.

### Mesenchymal stem cells isolation and expansion

First, tissue was separated and washed with the phosphate-buffered saline (PBS). It was minced into the small pieces measuring 1 mm3 and digested with 1 mg/ml collagenase type I (Sigma, Germany) for 60 minutes at 37˚C and centrifuged for 10 minutes at 500 g. Second, cells were plated in the 25 cm^2^tissue culture using Dulbecco’s Modified Eagle’s Medium (DMEM, Biowest, France) supplemented with 20% fetal bovine serum (FBS, Gibco, USA), 50 mg/ml of streptomycin and 50 U/ml of penicillin (Invitrogen, USA) at 37˚C in 95% air and 5% CO_2_. After that, when cultures reached at 80 to 90% confluence, eMSCs were trypsinized using trypsin EDTA 0.25% (Biowest, France) and then the media were replaced. For this study cells were treated with lovastatin and decitabin at the passage four.

### Flow cytometry analysis

To characterize and quantify the expression of MSCs markers according to the surface molecular markers (34), flow cytometry analysis was performed. First, cells were detached with trypsin EDTA 25% at the end of third passage and washed with PBS by centrifugation (300 g, 5 minutes). After that, cells (1×10^6^cells) were incubated with the monoclonal antibodies (e.g. CD90, CD44, CD146, CD45 and CD34) and the matched-isotype control for 30 minutes at 4˚C. Finally, cell analysis was performed using Partec CyFlow® Space flow cytometer system (German Biotechnology Company, Germany) and the flowmax Software.

### Osteogenic and adipogenic differentiation of endometrial mesenchymal stem cells

In order to perform the osteogenic and adipogenic differentiation, eMSCs were seeded at the density of 2×10^4^cells/cm_2_ in 24-well tissue culture plates and incubated in DMEM overnight at 37˚C and 5% CO_2_ until 80% confluency. Differentiation was carried out using osteogenic and adipogenic media according to the manufacturer’s instructions. In this respect, osteogenic differentiation was induced using DMEM high glucose supplemented with 10% FBS, 10 nM dexamethasone, 10 mM β-glycerophosphate and 10 μM ascorbic acid 2-phosphates (both from Sigma) for 21 days. Additionally, adipogenic differentiation was carried out by culturing eMSCs in DMEM high glucose supplemented with 10% FBS, 1 µM dexamethasone, 10 µM ascorbic acid 2-phosphate and 200 μM indomethacin (both from Sigma) for 21 days. Three weeks later, osteogenic and adipogenic differentiations were confirmed by Alizarin Red and Oil Red (both from Sigma) staining, respectively (35).

### MTT assay

First, eMSCs derived from the endometriotic tissues were seeded at the density of 1×10^4^cells/cm_2_ in a 24-well plate and cultured for 24 hours. Second, cells were treated with 1, 2 and 5 µM lovastatin diluted in dimethyl sulfoxide (DMSO), for 72 hours. Then, eMSCs were incubated with standard medium containing 3-(4,5-dimethylthiazol-2-yl)-2,5- diphenyltetrazolium bromide (Sigma) with final concentration of 0.5 mg/ml (stock solution 5 mg/ml MTT in PBS) for 4 hours at 37˚C. At the end of experiment, the medium was removed and 500 μl DMSO was added. Absorbance was evaluated at 540 nm in a 96-well plate using an Anthos 2020 Microplate Readers (Austria). Experiments were carried out in triplicate, from three independent experiments (36, 37).

### Treatment of endometrial mesenchymal stem cells with lovastatin and decitabin

First, MSCs from the human endometrium and endometriosis were seeded at an initial density of 60% confluence. They were then allowed to be attached overnight, and after that treated with lovastatin and decitabin (both from Sigma). According to the previous investigations (33, 38), MSCs were treated in the 1, 2 and 5 µM concentration of lovastatin for 72 hours, while these cells were treated in DMSO, as vehicle group. In addition, dose of 2 µM was used for decitabin treatment in the MSCs for 72 hours (39). After treatment, the cells were trypsinized and used for flow cytometry analysis, real- time polymerase chain reaction (PCR) and quantitative methylation specific PCR (qMSP).

### RNA extraction and quantitative analysis by real-time polymerase chain reaction

First, total RNA was isolated from the eMSCs with High Pure RNA Isolation Kit (Roche, Germany) according to the manufacturer’s protocol. Second, the purity of RNA was determined, by gel electrophoresis, photospectrometrically (ratio 260/280 nm), and by RT-PCR reactions. For each sample, 1 µg of RNA was used to generate cDNA with RevertAid First Strand cDNA Synthesis Kit (Thermo Scientific, USA). Then, quantitative reverse transcriptase PCR was carried out to determine the expression of genes encoding Bone Morphogenetic Protein2 (*BMP2*), GATA binding protein 2 (*GATA2*), Runt-related transcription factor (*RUNX2*), hypoxanthine phosphoribosyl transferase 1 (*HPRT1*) with StepOne™ Real-Time PCR system (Applied Biosystems, USA). Primers used for SYBR Green assay were: 

BMP2

F: 5ˊ-CCACCATGAAGAATCTTTGGAAGAAC-3ˊ

R: 5ˊ-TGATAAACTCCTCCGTGGGGA-3ˊ

GATA2

F: 5ˊ-GCTCGTTCCTGTTCAGAAGGC-3ˊ

R: 5ˊ-CCCATTCATCTTGTGGTAGAGGC-3ˊ

RUNX2

F: 5ˊ-CCCCACGACAACCGCACCAT-3ˊ

R: 5ˊ-CGCTCCGGCCCACAAATCTC -3ˊ (40)

HPRT

F: 5ˊ-GGTCCTTTTCACCAGCAAGCT-3ˊ

R: 5ˊ-TGACACTGGCAAAACAATGCA-3ˊ. 

*HPRT* values were used for normalization. PCR product length for *BMP2*, *GATA2*, *RUNX2* and *HPRT* primers was 101, 126, 289, and 94 bp, respectively. Gene expression was calculated using the ΔΔCt method (41). 

### Sodium bisulfite treatment of genomic DNA

First, DNA was isolated from eMSCs using High Pure PCR Template Preparation Kit (Roche) as recommended by the manufacturer’s instruction. Second, for sodium bisulfite treatment, 300 ng of DNA was denatured by 0.2 M NaOH for 10 minutes at 37˚C in 50 ml total volume. Then, 30 µl of freshly prepared 10 mM hydroquinone (Merck, US) and 520 μl of 3.5 M sodium bisulfite (pH=5, Merck, US) were added to the samples. After that, each DNA sample was incubated at 50˚C for 16 hours. Samples were also purified with Roche DNA purification columns based on the manufacturer’s instruction and eluted in 200 μl of elution buffer. At last, samples were desulfonated by 0.3 M NaOH treatment for 5 minutes at 20˚C. Finally, after ethanol precipitation, DNA was dissolved in 30 μl distilled water (42).

### Quantitative Methylation Specific polymerase chain reaction

For analyzing *BMP2* promoter methylation, MethySYBR Method was performed with StepOne™ Real-Time PCR System (Applied Biosystems). In this study, according to the one-step MethySYBR method (43), the primers (BMP2-EXT-F and BMP2-EXT-R; product length=308) were used in the externally nested real time PCR amplified the target gene regardless of their methylation status. This was used as a reference control to normalize the proportion of methylated target alleles which were detected by the methylation specific primer pair (BMP2-FM and BMP2-RM, product length=113) between the samples. Each reaction contained 20-25 ng of bisulfite-treated DNA as a template, 10 ml 2x RealQ Master Mix ampliqon and 500 nM of each forward and reverse primer (Table 1) in a total volume of 20 µl. For *BMP2*, real-time PCR thermocyclic conditions included an initial step of 10 minutes at 95˚C, followed by 40 cycles of 95˚C for 15 seconds, and 60˚C for 30 seconds. 

In this method, plasmid template was included as the control for calculation of methylation percentage of each sample. Methylated DNA level was calculated with 2^-ΔΔCt^ in which ΔΔC_t_ equals to ΔC_t sample_-ΔC_t plasmid_ (43). Furthermore, to evaluate the methylation status of *GATA2*, we performed qMSP using the primers directed against differentially methylated regions of exon 4 of *GATA2*. Briefly, primers were designed to determine either the methylated or unmethylated form of the sequence after the bisulfite converted sequences of the sense strand. Primer information is provided in Table 1. For *GATA2*, the thermocyclic conditions of real-time PCR included an initial denaturation step of 10 minute at 95˚C, followed by 40 cycles of 95˚C for 15 seconds and 57˚C for 30 seconds. Additionally, the product length for the *GATA2*-Meth and *GATA2*-Unmeth primers was 139 bp.

### Statistical analysis

Comparison of gene expressions, methylation values as well as cell viability tests between samples were assessed with a two tailed student’s t test using GraphPad Prism 6 software. Results were statistically significant at a P<0.05.

## Results

### Isolation and characterization of endometrial mesenchymal stem cells 

**Fig.1:**
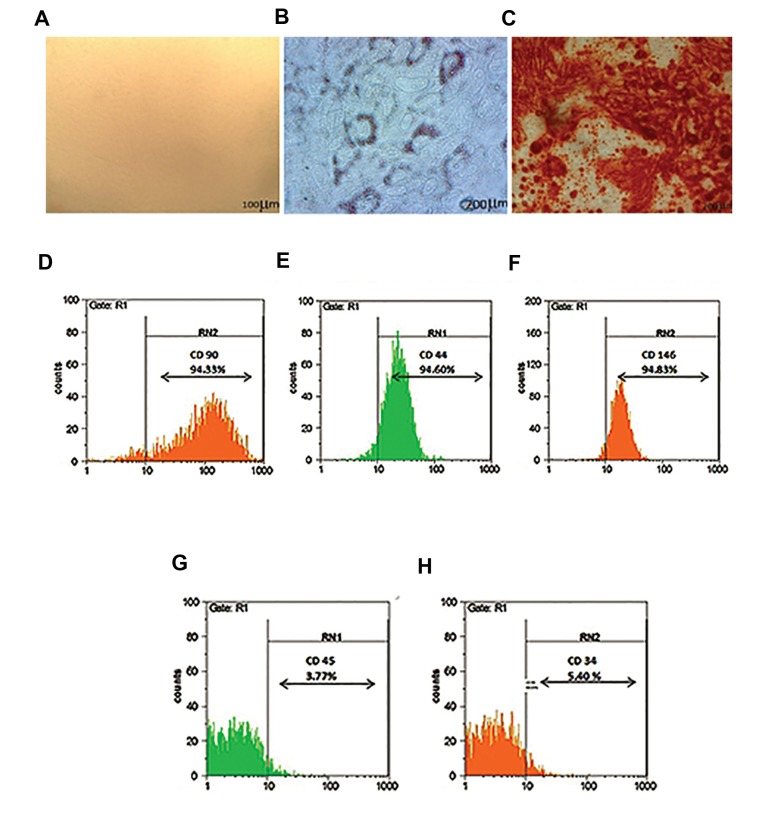
Mesenchymal stem cells (MSCs) characterization. Human endometrial MSCs (eMSCs) exhibited, A. A fibroblast-like cell shape. These cells also represented successful, B. Adipogenic, C. Osteogenic differentiation potential, showing a positive signal for D. CD44 (94.60%), E. CD90 (94.33%), F. CD146 (94.83%) and no signal for G. CD45 (3.77%), and H. CD34 (5.40%) markers (n=3).

MSCs from human endometrium were isolated and cultured, while they predominantly had fibroblastic shape as expected (Fig.1A). To evaluate differentiation potential of eMSCs, induction to adipogenic and osteogenic lineage was performed *in vitro*. A potential for the differentiation to adipogenic lineage was confirmed through staining of lipid vacuoles by oil red (Fig.1B). Furthermore, osteogenic differentiation was detected through alizarin red staining of calcium deposits (Fig.1C). Flow cytometer analysis indicated that cells expressed the mesenchymal markers CD44 (94.60%), CD90 (94.33%) and CD146 (94.83%), but they lacked hematopoietic markers including CD45 (3.77%) and CD34 (5.40%) (Fig.1D-H).

**Table 1:** Primer sequences for qMSP analysis of *GATA2* and *BMP2*


Gene name	Primer sequences (5ˊ-3ˊ)

*BMP2*-EXT	F: GTGTATTGGAGTAAGGTAGAGTGATG
	R: CCCAACCAAATACTAACACACAACAAC
*BMP2*-FM	F: GGTTGTTTCGAGTTATGGGTCGC
	R: AAAACCAACGCCCGAAAAACGCG
*GATA2*-Ex4-Meth	F: TTCGCGTAGTTGTTGTTTTTAGAC
	R: GAACCCAATACTCACCGTACG
*GATA2*-Ex4-Un	F: TTGTGTAGTTGTTGTTTTTAGATGA
	R: ACAAACCCAATACTCACCATACAC


### Lovastatin mediates up-regulation of *BMP2* and *RUNX2*

**Fig.2:**
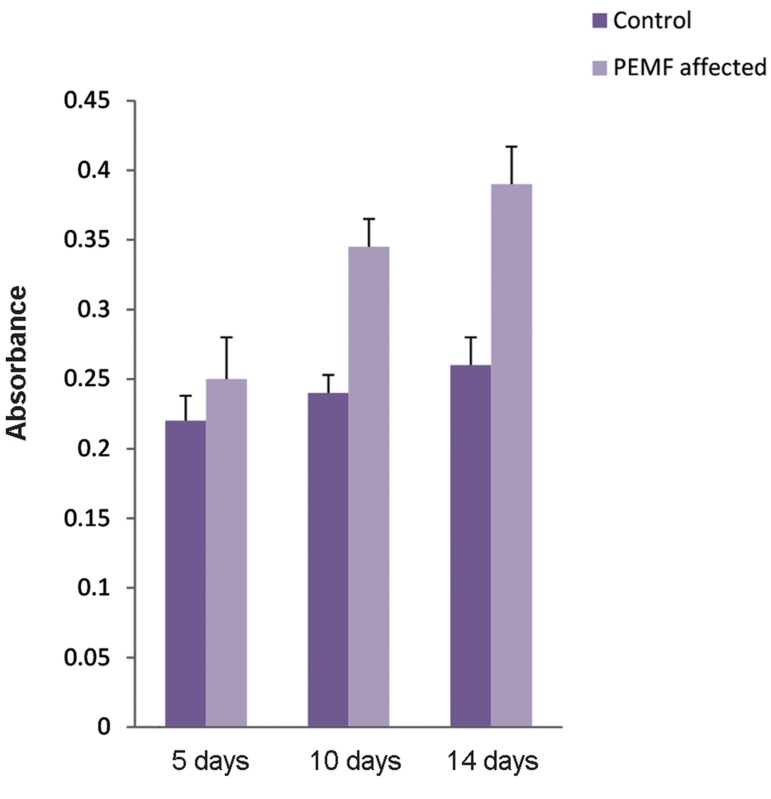
*BMP2* and *RUNX2* expressions following the lovastatin treatment in endometrial mesenchymal stem cells (eMSCs) cultures of three patients and three normal individuals, detected by quantitative real-time polymerase chain reaction (RT-PCR). A. Relative expression of *BMP2* at 2 μM statin-treated eMSCs was 1.69 ± 0.26 and 2.22 ± 0.1 fold higher than those of control eMSCs in patient and normal groups, respectively (P<0.05, Student’s t test) and B. *RUNX2* expression was up-regulated in plates treated with 2 and 5 µM of lovastatin as well as 2 µM decitabin in comparison with untreated control of patient group (2.58 ± 0.32 fold, 1.86 ± 0.22 fold and 2.26 ± 0.18 fold respectively, P<0.05 for 2 and 5 µM of lovastatin and P<0.01 for 2 µM decitabin) and normal group (3.35 ± 0.21 fold, 2.02 ± 0.10 fold and 2.12 ± 0.10 fold respectively. *; P<0.05 and **; P<0.01 in comparison to untreated control in each groups.

To investigate the effect of different lovastatin concentrations on *BMP2* and *RUNX2* mRNA expression, eMSCs were isolated from the patient and normal individuals and then were incubated for 72 hours with 2 and 5 µM concentrations of lovastatin. Relative expression amounts of *BMP2* in plates treated with the different concentrations of lovastatin, vehicle (DMSO) and 2 µM decitabin, as the positive control, are shown in Figure 2. In comparison with untreated control of both groups, relative expressions of *BMP2* at 2 µM lovastatin-treated eMSCs were 1.69 ± 0.26 and 2.22 ± 0.1 fold further than those of control eMSCs in the patients and normal groups, respectively. Statistical analysis showed a significant difference between 2 μM lovastatin and control in both groups (P<0.05, Student’s t test). Furthermore, *RUNX2* expression was markedly up-regulated in the plates treated with 2 and 5 µM of lovastatin as well as 2 µM decitabin in comparison with the untreated control of patient group (2.58 ± 0.32 fold, 1.86 ± 0.22 fold and 2.26 ± 0.18 fold, respectively, P<0.05 for 2 and 5 µM of lovastatin and P<0.01 for 2 µM decitabin). *RUNX2* expression was also up-regulated in plates treated with 2 and 5 µM of lovastatin as well as 2 µM decitabin compared to the untreated control of normal group (3.35 ± 0.21 fold, 2.02 ± 0.10 fold and 2.12 ± 0.10 fold respectively, P<0.05).

### Lovastatin mediates down-regulation of *GATA2*

**Fig.3:**
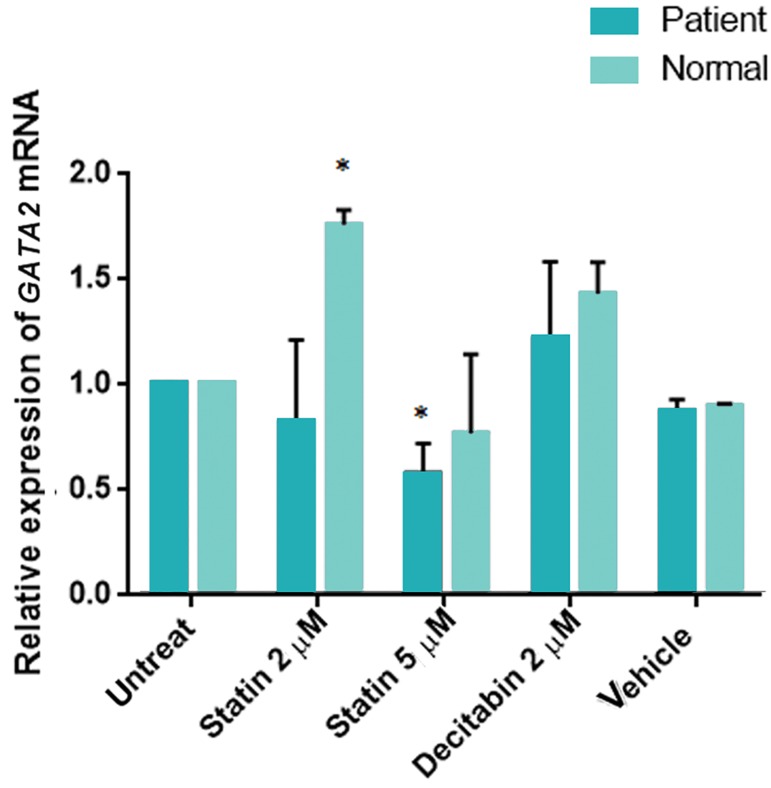
*GATA2* expression following the lovastatin treatment in endometrial mesenchymal stem cells (eMSCs) cultures of three patients and three normal individuals, detected by quantitative real-time polymerase chain reaction (RT-PCR). Relative expression of *GATA2* was decreased in response to 5 μM lovastatin treatment in comparison with untreated control of patient gro0.57 ± 0.14 fold (P<0.05). On the other hand, *GATA2* expression at 2 μM lovastatin-treated eMSCs was 1.75 ± 0.07 fold higher than those of control eMSCs in normal. *; P<0.05 in comparison to untreated control in each groups.

Following 2 μM lovastatin treatment, *GATA2* expression was slightly down-regulated, while the expression of *GATA2* was significantly decreased in response to 5 μM lovastatin treatment in comparison with the untreated control of patient group (Fig.3, 0.57 ± 0.14 fold, P<0.05). On the other hand, *GATA2* expression at 2 μM statin- treated eMSCs were 1.75 ± 0.07 fold higher than those of the control eMSCs in normal (Fig.3, P<0.05). There was also no significant difference in the expression levels of *GATA2* in response to 2 µM decitabin, compared to the untreated control of both groups.

### Lovastatin treatment leads to epigenetic modification of the *BMP2* and *GATA2*

**Fig.4:**
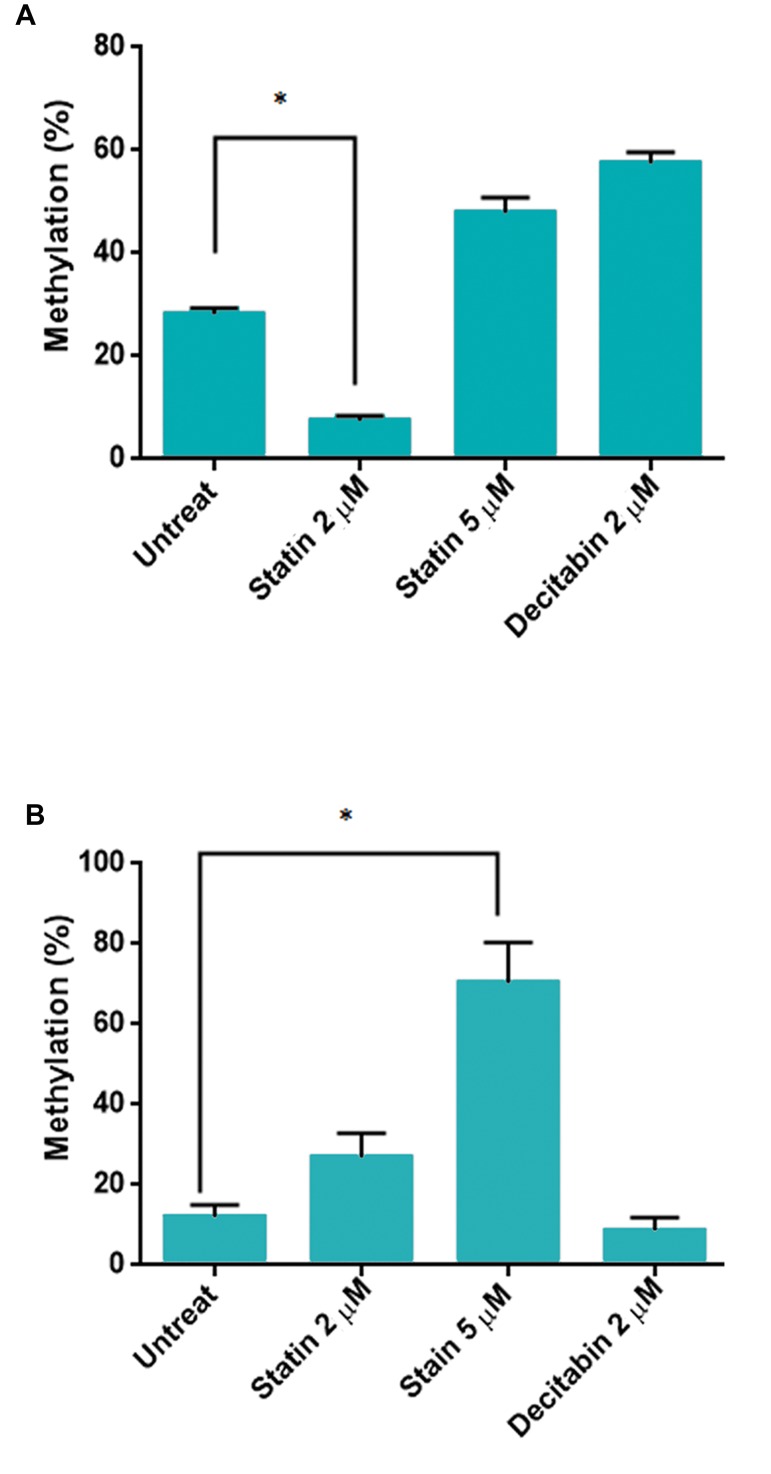
Quantitative methylation specific PCR (qMSP) analysis of *BMP2* promoter region and *GATA2* exon 4 in endometrial mesenchymal stem cells (eMSCs) treated with different concentration of lovastatin. A. qMSP results showed that *BMP2* promoter methylation was decreased from 28.2 to 7.6% after treatment with 2 μM of lovastatin for 72 hours (P<0.05, Student’s t test) and B. qMSP results showed that *GATA2* exon 4 methylation was increased from 12.0 to 26.95 and 70.49% after treatment with 2 and 5 μM of lovastatin for 72 hours, respectively. *; P<0.05 in comparison to untreated control in each groups.

To determine involvement of DNA methylation in the *BMP2* gene down-regulation of eMSCs treated with the different concentration of lovastatin, we used quantitative Methylation Specific PCR (qMSP) for the respective promoter status, starting 214 bp upstream of exon 1. This region contains a CpG island that methylated in the gastric and colorectal cancers (18, 44). As Figure 4A shows, lovastatin treatment induced demethylation of the *BMP2* promoter in eMSCs treated with 2 μM lovastatin for 72 hours. The qMSP results showed that *BMP2* promoter methylation was decreased from 28.2 to 7.6% in eMSCs after treatment with 2 μM lovastatin for 72 hours (P<0.05, Student’s t test).

We also performed qMSP for *GATA2* before and after lovastatin and decitabin treatments using the methylated and unmethylated primers directed against differentially methylated region of *GATA2* exon 4 (45). As Figure 4B shows, lovastatin treatment induced methylation of the differentially methylated region of *GATA2* exon 4 in eMSCs treated for 72 hours with 2 and 5 μM lovastatin. The qMSP results showed that the *GATA2* exon 4 methylation was increased in eMSCs from 12.0 to 26.95 and 70.49% after treatment with 2 and 5 μM lovastatin for 72 hours, respectively (P<0.05, Student’s t test).

### No influence on the cell viability by lovastatin up to 5 µM concentration

**Fig.5:**
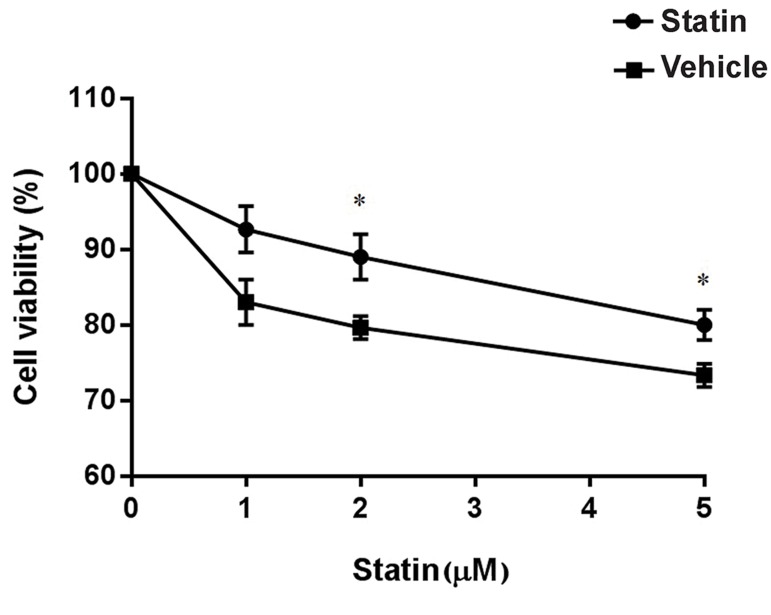
Mesenchymal stem cells (MSCs) were plated in 24 well plates and either no treated, or treated with dimethyl sulfoxide (DMSO) or 1, 2 and 5 μM lovastatin (diluted in DMSO) for 72 hours, followed by MTT test. Values are shown as living cells percentage relative to the control untreated cells with set at 100% in control values. Results expressed the mean ± SD (n=3). *; P<0.05 in comparison to untreated control in each groups.

Changes in the cell viability could lead to a decrease in cell number and a low influence on cell therapy. In order to determine the effects of lovastatin (diluted in DMSO) and DMSO on eMSCs viability, cells were incubated with 1, 2 and 5 µM lovastatin for 72 hours, and then, mitochondrial dehydrogenase activity was evaluated in the living cells by the MTT test (46). The cell viability in vehicle as well as treatment group was observed in approximately 80% of cells in comparison to the untreated group. However, statistical analysis showed a significant difference at the enzyme level under 2 and 5 µM lovastatin and DMSO treatment (Fig.5, P<0.05, Student’s t test).

### Lovastatin down-regulates the melanoma cell adhesion molecule

**Fig.6:**
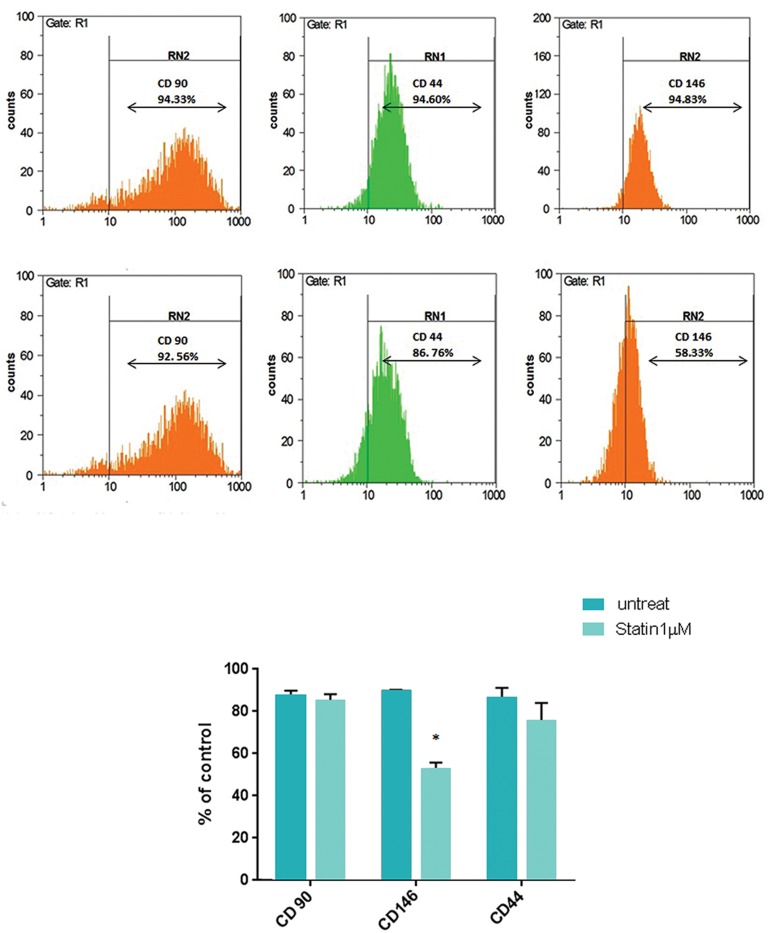
Flow cytometry analysis of eMSCs markers treated with 1 µM lovastatin. The results indicated that CD146 cell marker was down-regulated to 53% in response to 1 μM lovastatin in comparison with untreated group. Data are from three experiments ± SD (n=3). *; P<0.05 in comparison to untreated control.

To investigate the effect of lovastatin on eMSCs markers, cells were treated with 1 μM lovastatin for 72 hours and then, analyzed by flow cytometer. The results indicated that CD146 cell marker was down-regulated to 53% in response to 1 µM lovastatin, compared to the untreated group (Fig.6, P<0.05, Student’s t test). In this respect, recent studies have shown that Melanoma Cell Adhesion Molecule (MCAM/ CD146) was a key marker of endometrial stem/ progenitor cells involved in the inflammation and angiogenesis procedures (47, 48).

## Discussion

Stem cell theory began the final advanced avenue for the etiology of endometriosis. A great number of studies demonstrated presence of the endometrial stem cells, not only from residing cells in the endometrium but also from reprograming bone marrow MSCs (17). Feasibility of targeting stem cells was suggested to be as of the remarkable advancement to eliminate endometriosis (49). 

This study evaluated the effect of lovastatin on eMSCs properties including differentiation and proliferation potential. In the current experiment, *BMP2* activity was significantly augmented in eMSCs within three days after treatment with 2 µM concentration of lovastatin. *BMP2* activity was proved to be a marker of osteogenesis differentiation (38). Previous studies have demonstrated that lovastatin increased the level of *BMP2* gene expression (50,51). Moreover, *BMP2* reportedly was downregulated in endometriosis (29). In this respect, there was remarkable evidence showing that *BMP2* signaling pathway plays a pivotal role in the decidualization (52,53). The study carried out by van Kaam et al. (54) revealed that both ectopic and eutopic endometrium of patients suffering from endometriosis demonstrated a decreased capacity for differentiation, as well as decidualization and implantation. 

In addition, *RUNX2* expression level was significantly increased in the treatment of 2 and 5 μM lovastatin, compared to the control group. *RUNX2* is a major downstream mediator of *BMP2*, functioning and playing a critical role in the stromal differentiation and decidualization (55). Furthermore, MSCs differentiation towards osteogenic lineage was determined by definite group of elements (56). Among these factors, the first and highly specific marker was *RUNX2*. In this line, *RUNX2* activated osteogenic differentiation by signaling pathways including *BMP2* and TGF-β1 (57,58). 

In the present study, expression of GATA2 was investigated in eMSCs after lovastatin treatment. In this case, *GATA2* mRNA level was significantly decreased in response to the 5 μM lovastatin treatment, compared to the untreated control of patient group. 

Increasingly, it was found that *GATA2* expression, a member of the six zinc-finger family transcription factors, was essential for various tissues including urogenital and hematopoietic system and adipose maturation (59). Moreover, Kamata et al. (32) demonstrated that *GATA2* could be one of the significant factors regulating differentiation of bone marrow MSCs toward adipocytes. Given the results of previous investigations (59,32), reduction of *GATA2* in response to lovastatin treatment might be in favor of decreased proliferation and increased differentiation potentials. However, this observation should be confirmed by other studies. 

Furthermore, SYBR Green-based quantitative real time PCR method was performed to analyze DNA methylation level in eMSCs. The MethySYBR assay is a very sensitive, precise and less vulnerable to false positives (60). In this study, lovastatin treatment induced DNA demethylation and reactivation of *BMP2* gene expression, which was suppressed by hypermethylation in the endometriosis. More importantly, we found demethylation of other methylated genes including *RUNX2* in the endometriosis after treatment with lovastatin, implying more general effect on gene hypermethylation. Given the results of this study, it is not obvious how lovastatin inhibits DNMTs. Kodach et al. (18) showed that lovastatin has either little or no effect on DNMTs expression levels. Therefore, further investigations are required to evaluate the mechanism(s) by which lovastatin inhibit DNMTs. On the other hand, we found that lovastatin induced methylation of *GATA2* factor. This result was in consistent with the latest data reported by MacLeod et al. (20), showing that lovastatin therapy is related to higher MTHFR methylation levels in a stroke group implying that statins can induce DNA methylation. 

We also evaluated the effect of lovastatin on expression of MSCs markers. Some recent investigations have indicated that CD146 could be considered as a highly specific marker of endometrial stem/progenitor cells (17,61). In addition, Figueira et al. (13) used CD146 marker to identify mesenchymal stem cells for the first time. The eMSCs expressed typical MSC surface markers including CD44, CD90 and CD105. 

In this study, flow cytometer data displayed that CD146 was reduced in response to lovastatin treatment in eMSCs, suggesting that CD146 could effectively be implicated in the endometriosis pathogenesis by activating the angiogenesis and inflammation (47). Additionally, CD146 is an endothe¬lial cell adhesion molecule that is upregulated in different types of malignant cell, such as ovarian cancer (62,65). A great number of experiments have suggested that CD146 induced angiogenesis, tumor growth and metastasis (66). Moreover, Flanagan et al. (67) showed that laminin-411 attached to CD146 enabling TH17 cell penetrate into the tissues and induce inflammation. Studies have revealed that lovastatin, which is a potent inhibitor for the expression of VEGF, plays a pivotal role in diminishing blood-vessel formation (68). Similarly, in the recent study, Jiang et al. (69) reported that CD146 interacts with VEGFR-2 in a tumor angiogenesis mechanism. In line with previous investigations, our research presents a new target of action for lovastatin, in inhibition of angiogenesis via suppressing CD146. 

Based on the previous *in vitro* studies, doses of 1 to 5 µM were used for lovastatin treatment in MSCs (33,38). In these experiments, mild growth stimulatory effects in eMSCs were derived from human endometrium, and endometriosis was observed at dose of 1 and 5 µM. In addition, Kupcsik et al. (38) revealed that lovastatin concentration at 10 µM is associated with cytotoxic effects and leads to detachment of eMSC from culture plate. 

Zhou and Hu (23) showed that stem cell differentiation could be augmented by DNA demethylation, starting advancement for studying the induction of stem cell fate through epigenetic reprograming. In this study, for the first time, we demonstrated that aberrant demethylation of CpG island promoter of *BMP2* occurred in endometriosis tissues. We also provided a facet of molecular basis of the *BMP2* down-regulation in these tissues from the viewpoint of epigenetic disease. It is hoped that epigenetic reprograming of *BMP2* becomes a helpful cue for the further research in the pathogenesis of endometriosis. 

## Conclusion

The proposed mechanisms of statins action on the endometriosis tissues are suppression of endometrial cells proliferation and apoptosis, reduction of oxidative stress and inflammation, and inhibition of the angiogenesis. Our study indicated that lovastatin treatment could increase osteogenic differentiation through up-regulation of *BMP2* and *RUNX2* mRNA expression. In addition, reduction of *GATA2* in response to lovastatin treatment might be in favor of increased adipogenic differentiation potentials. Expression of stem cell markers and subsequently stemness was also reduced in the eMSCs after lovastatin treatment. 

Furthermore, consistent with the previous studies, our investigation revealed that lovastatin decreased angiogenesis and increased implantation and decidualization. 

Several recent investigations have suggested that statins could have a pivotal role in the medical management of women suffering from endometriosis. They also offer clinical benefits without interfering in estrogen. Despite this fact, more clinical trials are needed to confirm the safety and effectiveness of this kind of treatment in endometriosis. 
